# Electroacupuncture-driven endogenous circulating serum exosomes as a potential therapeutic strategy for sepsis

**DOI:** 10.1186/s13020-023-00816-7

**Published:** 2023-08-27

**Authors:** Jingyu Zhang, Meijuan Wang, Xiyou Hu, Ningcen Li, PeiYong Loh, Yinan Gong, Yong Chen, Lifen Wang, Xiaowei Lin, Zhifang Xu, Yangyang Liu, Yi Guo, Zelin Chen, Bo Chen

**Affiliations:** 1 Research Center of Experimental Acupuncture Science, Tianjin University of Traditional Chinese Medicine, Tianjin, 301617 People’s Republic of China; 2 Tianjin Key Laboratory of Modern Chinese Medicine Theory of Innovation and Application, Tianjin University of Traditional Chinese Medicine, Tianjin, 301617 People’s Republic of China; 3 School of Acupuncture & Moxibustion and Tuina, Tianjin University of Traditional Chinese Medicine, Tianjin, 301617 People’s Republic of China; 4 School of International Education, Tianjin University of Traditional Chinese Medicine, Tianjin, 301617 China; 5 National Clinical Research Center for Chinese Medicine Acupuncture and Moxibustion, Tianjin, 300381 People’s Republic of China; 6 Tianjin Binhai New Area Hospital of Traditional Chinese Medicine, Fourth Teaching Hospital of Tianjin University of Traditional Chinese Medicine, Tianjin, 300451 People’s Republic of China

**Keywords:** Electroacupuncture, Sepsis, Serum exosomes, Survival rate, Inflammatory cytokines, In vivo imaging of small animals, MiRNA sequencing, Network biology analysis

## Abstract

**Background:**

Sepsis poses a serious threat to human life and health, with limited options for current clinical treatments. Acupuncture plays an active role in treating sepsis. However, previous studies have focused on the neuromodulatory effect of acupuncture, neglecting its network modulatory effect. Exosomes, as a new way of intercellular communication, may play an important role in transmitting acupuncture information. This paper explores the possibility of electroacupuncture-driven endogenous circulating serum exosomes and their carried miRNAs as a potential treatment for sepsis.

**Methods:**

The sepsis mouse model was established by intraperitoneal injection of lipopolysaccharide (LPS) (12 mg/kg, 24 mg/kg), and EA (continuous wave, 10 Hz, intensity 5) or intraperitoneal injection of Acupuncture Exosomes (Acu-exo) were performed before the model establishment. The therapeutic effect was evaluated by survival rate, ELISA, H&E staining and lung wet/dry weight ration (W/D). In vivo imaging of small animals was used to observe the accumulation of Acu-exo in various organs of sepsis mice. LPS was used to induce macrophages in cell experiments, and the effect of Acu-exo on macrophage inflammatory cytokines was observed. In addition, The miRNA sequencing method was further used to detect the serum exosomes of normal and EA-treated mice, and combined with network biology analysis methods to screen possible key targets.

**Results:**

EA and Acu-exo reduced the W/D and lung tissue damage in sepsis mice, down-regulated the expression of serum inflammatory cytokines TNF-α and IL-6, and increased the survival rate of sepsis mice. In vivo imaging of small animals found that Acu-exo were accumulated in the lungs of sepsis mice. Cell experiments proved that Acu-exo down-regulated the expression of inflammatory cytokines TNF-α, IL-6 and IL-1β to alleviate the inflammatory response induced by LPS in macrophages. MiRNA sequencing revealed 53 differentially expressed miRNAs, and network biology analysis revealed the key targets of Acu-exo in sepsis treatment.

**Conclusion:**

Electroacupuncture-driven endogenous circulating serum exosomes and their carried miRNAs may be a potential treatment for sepsis.

## Introduction

Sepsis is defined as life-threatening organ dysfunction caused by a dysregulated host response to infection [1]. Annually, there are 30 million new sepsis patients in the world, and more than one-fifth of them die. In 2017, the number of global deaths on sepsis accounted for 19.7% of overall deaths in the world [2]. Another study showed that the incidence of sepsis among hospitalized patients was 189 cases per 100,000 people per year, with a case fatality rate of 26.7%. The incidence rate of ICU inpatients was 58 cases per 100,000 people per year, with a case fatality rate of 41.9% [3]. The high morbidity and mortality of sepsis impose a heavy burden on society and families. In the treatment of excessive inflammation, immunosuppression, and organ dysfunction, much exploration has also been carried out, such as the application of glucocorticoids and immunosuppressants, as well as protective treatment of organ function. However, these symptomatic and supportive treatments did not significantly reduce patient mortality, and even increased the risk of drug resistance [4]. Therefore, there is an urgent need to develop new treatments.

Acupuncture therapy have been continuously developed through thousands of years of clinical practice, and are widely used in nearly 200 countries [5]. A systematic review of acupuncture in the treatment of sepsis (20 RCTs, 1337 patients) showed that acupuncture combined with conventional therapy can significantly reduce 28-day mortality and shorten the length of ICU stay. It can improve gastrointestinal function in patients with sepsis, reduce the expression of procalcitonin and TNF-α [6]. Some progress has been made in recent years regarding the mechanism of acupuncture for treating sepsis. Acupuncture stimulation can drive various somatosensory-parasympathetic pathways in an acupoint- and intensity- dependent manner [7]. Studies in 2014 and 2020 have found that the anti-sepsis effect of EA is related to the vagus nerve-adrenal medulla-dopaminergic anti-inflammatory pathway, which explain the neuroendocrine regulation pathway of EA and its anti-inflammatory effects [8, 9]. Further research in 2021 revealed that DRG sensory neurons, marked by PROKR2-Cre, specifically innervate the deep fascia tissues of the limbs, such as periosteum, articular ligaments, and myofascia. The activation of the vagus nerve-adrenal anti-inflammatory pathway through low-intensity EA stimulation requires DRG sensory neurons, providing a modern neuroanatomical basis for the relatively specific existence of acupoints [10]. However, most current studies on the mechanism of acupuncture anti-inflammation focus on neural pathways, while little attention has been paid to network regulation. The treatment of diseases by acupuncture is not done by a single signal molecule, signal pathway, nervous system, endocrine system or immune system alone. Acupuncture plays an overall network regulation role by stimulating the body’s neuroendocrine immune system [11].

Exosomes have many natural advantages that enable them to efficiently deliver various proteins and genes to target cells, making them an effective therapeutic vesicle [12–14]. Exosomes participate in the body’s neuronal communication, antigen presentation, immune response, control of aging, cell proliferation and other biological processes in the nervous, endocrine, and immune systems. Therefore, exosomes are considered important carriers of intercellular communication [15, 16]. Our previous research found that the exosome antagonist GW4869 can reverse the efficacy of EA in treating sepsis, suggesting that exosomes play an important role in EA effect. Therefore, we believe that exosomes may be an important carrier of transmitting acupuncture information [17–19]. This study further explores the role of Acupuncture Exosomes (Acu-exo) and their carried miRNAs in sepsis treatment, which may provide the possibility for the clinical translation of acupuncture.

## Experimental materials and methods

### Laboratory animals

All procedures were carried out strictly in accordance with the recommendations in the Guide for the Care and Use of Laboratory Animals of the National Institutes of Health. We selected healthy male C57BL/6 J mice aged 6–8 weeks with a body weight of (20 ± 2) g. These mice were purchased from Beijing SiPeiFu Laboratory Animal Technology Co. Ltd [License No. SCXK (Jing) 2019–0010]. The pellet feed was provided by the Experimental Animal Center of Tianjin University of Traditional Chinese Medicine, and the experiment was carried out after 5 days of adaptive rearing in the same standard environment. Individual mice are considered as the experimental units in the study, and random numbers are generated by the standard “ = RAND ()” function in Microsoft Excel.

### Establishment of a mouse model of sepsis

Mice were treated with intraperitoneal injection (i.p.) of lipopolysaccharide (LPS) (Escherichia coli 0111: B4, L2630, Sigma, USA) (12 mg/kg, 24 mg/kg) to establish a model of sepsis. The sepsis mice exhibited decreased activity, lethargy, piloerection, periocular white discharge, and tremors (refer to Murine sepsis score scale) [20]. However, if the mouse dies, neither the serum nor the tissue will be collected. Mice in the control group were intraperitoneally injected with the same dose of phosphate buffered saline (PBS), which is the solvent used to dissolve LPS. The observation of the survival rate started after the injection of LPS. The survival of the mice was observed every 2 h for the first 3 days (72 h), and then every 6 h from the 4th day to the 7th day (72 h ~ 168 h). The time of death for the mice is recorded within seven days (168 h).

### Treatment methods

Zusanli acupoint (ST36) was located with reference to “Names and Locations of Acupoints Commonly Used in Laboratory Animals Part 3: Mice” [21], at the posterolateral side of the knee joint, about 2 mm below the capitellum of the fibula, and the penetration depth was 3 ~ 5 mm. The instrument used for EA is“The HuaTuo electronic acupuncture treatment instrument (SDZ-V)”. The parameters of electroacupuncture pretreatment were continuous wave, 10 Hz, intensity 5, for 15 min. The specification of acupuncture needle is 0.25 × 13 mm (ZHONGYANTAIHE, Beijing, China). Acu-exo (i.p.) was extracted from the circulating serum of normal mice that had undergone EA for different days.

### Enzyme-linked immunosorbent assay (ELISA)

Blood was collected from the eyes of mice. All reagents and samples were taken out of the refrigerator in advance and allowed to equilibrate at room temperature for 30 min. TNF-α, IL-6, and IL-1β expression levels in animal serum or cell supernatant were measured using an ELISA kit (Cat No. SBJ-M0030, SBJ-M0657, SBJ-M0027, SenBeiJia, Nanjing, China) following the manufacturer’s instructions.

### Histological observation

The tissues were fixed in paraformaldehyde, embedded in paraffin, and then sliced into sections. Hematoxylin & eosin (H&E) staining was used to observe tissue damage. The results were observed using an optical microscope and images were collected (H&E staining results scanning instrument, APERIO VERSA 8, Leica, Germany; H&E staining results observation and photo apparatus BX51, Olympus, Japan).

### Lung wet/dry weight ration (W/D) measurement

The fresh lung tissues collected were weighed using a One-thousandth precision balance (WANT, Changzhou, China) and the wet weight (W) was recorded. The lung tissue was then placed in an Electric blast drying oven (YiHeng, Shanghai, Chian) at 80 °C for 24 h to complete dehydration. The dried lung tissue was weighed again and the dry weight (D) was recorded. The W/D represents the water content of the lung tissue.

### Serum exosomes harvest

Blood was collected from the eyes of mice. Any cells and cell debris are removed in serum in high-speed centrifugation (3000 g, 15 min, 4 °C). The supernatant was then transferred to a new tube and ExoQuick Precipitation agent was added (Cat No. EXOQ20A-1, SBI, USA, ratio 4:1) (4 °C, 30 min). After centrifugation (1500 g, 5 min, 4 °C), the supernatant was removed. The remaining precipitates were identified as exosomes.

### Transmission electron microscopy (TEM)

Isolated exosomes were fixed in paraformaldehyde, loaded onto formvar carbon-coated grids, and left to stand (15 min). The samples were then negatively stained with uranyl oxalate for 5 min and then visualized using a Hitachi H-600 transmission electron microscope (Japan).

### Western blot analysis (WB)

Proteins were extracted from exosomes using RIPA lysis buffer (Beyotime, China), and their concentrations were measured with BCA Protein Assay Kit. Samples with equal amounts of protein were separated by SDS-PAGE gels (Bio-Rad, USA), transferred to PVDF membranes (Chemicon/Millipore, USA), and used the indicated primary antibody overnight. Primary antibodies for CD9 (60232-I-Ig, Proteintech, Rosemont, IL), CD63 (sc-5275(M), Santa Cruz, CA, USA) and TSG101 (Abs115706, Absin, shanghai, China) (1:1500) were purchased from Abcam (USA). Secondary antibody (1:5000) was from Affinity (USA). Premixed chemiluminescent substrates were exposed to PVDF membranes.

### Nanoparticle tracking analysis (NTA)

The average number and size of exosomes were determined using zetaview (Particle Metrix, Germany). Open the inlet and outlet of the NTA instrument, and inject 10 ml of distilled water into the cell channel. Ensure that the exosomes being measured are free of air bubbles and do not inject air bubbles into the system. Immediately close the entrance. Click OK to execute the exosomes.

### In vivo imaging of small animals

The lipid bilayer of exosome was labeled with the DiR fluorescent dye (DiOC18) (UR21017, Umibio, Shanghai, China). The fluorescent agent DiR and DiR-stained exosome (DiR-Acu-exo) were injected into the tail vein with 60 μL each. The small animal in vivo optical imager (PerkinElmer, Shanghai, China) was used to image the heart, liver, spleen, lung, and kidney of a mouse after DiR and DiR-Acu-exo injection 2 h, 4 h at each time point. The fluorescence intensity was analyzed to observe the accumulation of exosomes in the organs.

### LPS-induced macrophage RAW264.7

Mouse macrophage RAW264.7 was donated by the School of Integrated Traditional Chinese and Western Medicine at Tianjin University of Traditional Chinese Medicine. Macrophages were cultured after configuring the culture medium according to the ratio of 90% DMEM cell culture medium (164210, Pricella, Wuhan, China) + 10% fetal bovine serum (164210, Pricella, Wuhan, China) + 1% double antibody solution (PB180120, Pricella, Wuhan, China). The number of cells in each group was 5 × 10^5^. After stimulating macrophages with LPS (10 μg/mL) for 24 h, Acu-Exo/NC-Exo (20 μg/mL) was added to co-culture for 24 h, and then the cell supernatant was collected for the detection of inflammatory factors.

### MiRNA sequencing for exosomes

Each exosome sample (~ 200 μL) was treated with 500 μL NucleoZOL reagent (Cat No. 740404, MN, Germany), and the total RNA was extracted according to the manual. Thermo Fisher Nanodrop 2000 and Qubit 3.0 RNA BR assay were used to analyze the total RNA yield. The sequencing libraries were constructed following Sample Preparation Guide of VAHTS Universal V6 Small RNA Library Kit. Polyacrymide PAGE Electrophoresis method was used for library size selection. The band (~ 140 bp) indicated miRNA library. The purified libraries were prepared for sequencing since passed quality control provided by Thermofisher Qubit 3.0 and Agilent 2100 Bioanalyzer. The miRNA libraries were sequenced on Illumina Novaseq 6000 sequencer using PE150 sequencing method. The data obtained by sequencing were screened for differentially expressed miRNAs according to the criteria of P < 0.05 and |Fold Change|> 2. Three databases of miRDB, miRTarBase and TargetScan were used to predict target genes. Then the target genes were subjected to KEGG enrichment analysis.

### Network biology analysis

The mouse differential target genes obtained by high-throughput sequencing were mapped to human genes (HGNC: https://www.genenames.org/) and compared with the human sepsis gene database (https://www.genecards.org; https://www.disgenet.org/; https://www.omim.org/) to screen out the potential targets of Acu-exo for treating sepsis. Then, the string data analysis platform (http://string-db.org/) was used to analyze the interaction genes, KEGG and genes-source of key targets in order to explore the possible biological mechanism of Acu-exo in treating sepsis. Finally, it was visualized using cytoscape software (3.9.0/Java 11).

### Statistical analysis

IBM SPSS Statistics 26 software and GraphPad Prism 8 software were used for statistical analysis of experimental data. The data in each group were tested for normality and homogeneity of variance. If the data in each group was normally distributed and had homogenous variance, they would be analyzed using one-way analysis of variation (ANOVA) followed by the Fisher’s Least Significant Difference (LSD) post hoc test. Otherwise, a nonparametric test would be used. Kaplan–Meier analysis and Log-rank test were used to perform survival analysis. The data were presented as mean ± SEM, and statistical significance was considered at *P* < *0.05*.

## Results

### EA increased the survival rate of sepsis mice

**Fig. 1:**
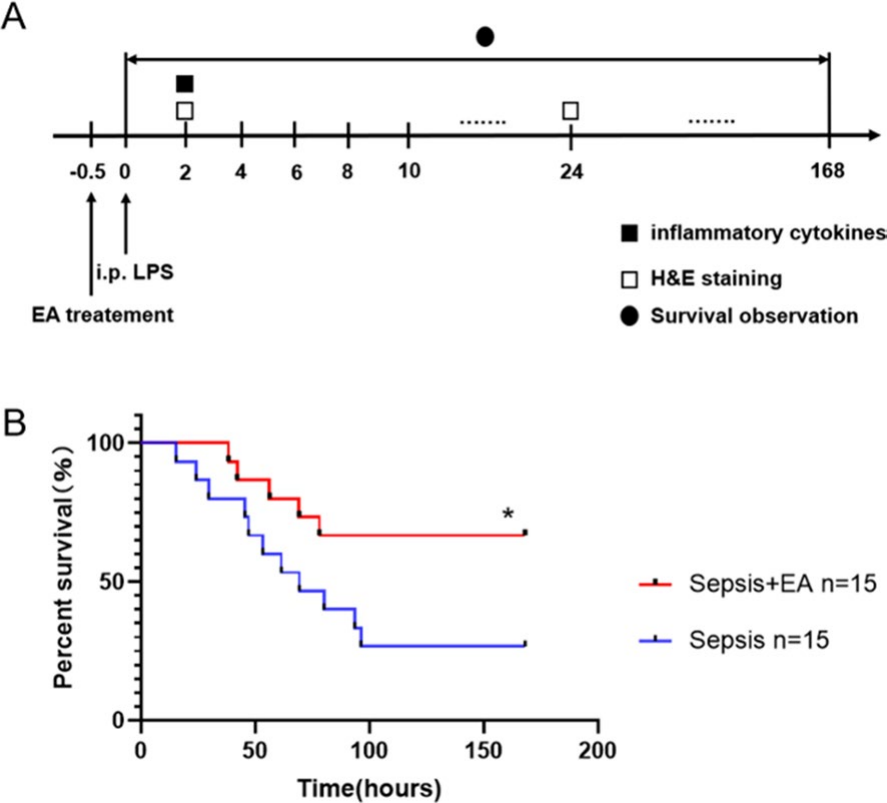
Effect of EA on the survival rate of sepsis mice. **A** Experimental procedures. **B** 7-day survival rate of LPS (12 mg/kg, i.p.) induced sepsis mice (n = 15/group). All data represent the mean ± SEM. *P < 0.05, compared with the sepsis group

We found that EA significantly increased the survival rate of sepsis mice from 26.67% to 66.70% (*P* < 0.05) (Fig. 1B).

### Characterization of exosomes extracted from C57b/6 J mice serum

**Fig. 2:**
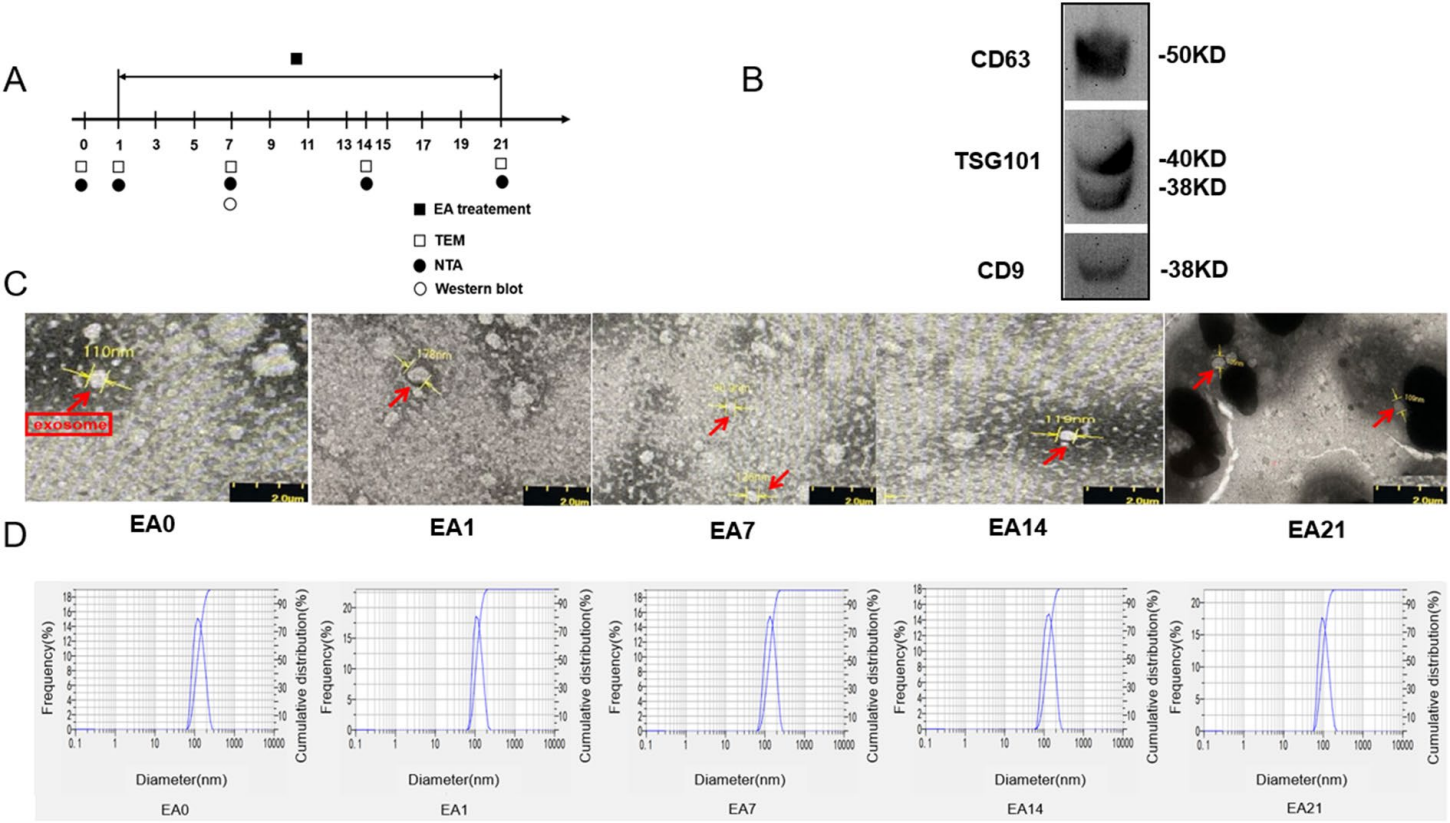
Markers, morphology and diameter of exosomes. **A** Experimental procedures. **B** WB was used to identify exosomes markers (CD9, CD63, TSG101), exosomes extracted from the circulating serum of normal mice treated with EA7. **C** TEM was used to observe exosome morphology. **D** NTA was used to measure the particle diameter of exosomes

Exosomes were isolated by ExoQuick kit and centrifugation. WB results showed that exosome markers (CD9, CD63, TSG101) extracted from the circulating serum of normal mice treated with electroacupuncture for 7 days (EA7) were positive (Fig. 2B). The exosomes extracted from the circulating serum of normal mice treated with EA1, EA7, EA14, EA21 and without EA treatment (EA0) were round or round-like (Fig. 2C). The particle size peaks of exosomes extracted from the circulating serum of normal mice treated with EA0, EA1, EA7, EA14, EA21 were 124.8 nm, 113.6 nm, 131.4 nm, 127.6 nm and 99.6 nm (Fig. 2D). These results indicate that we have successfully isolated exosomes from serum.

### Acu-exo’s time-efficacy relationship, concentration-efficacy relationship and accumulation in various organs in sepsis mice

**Fig. 3:**
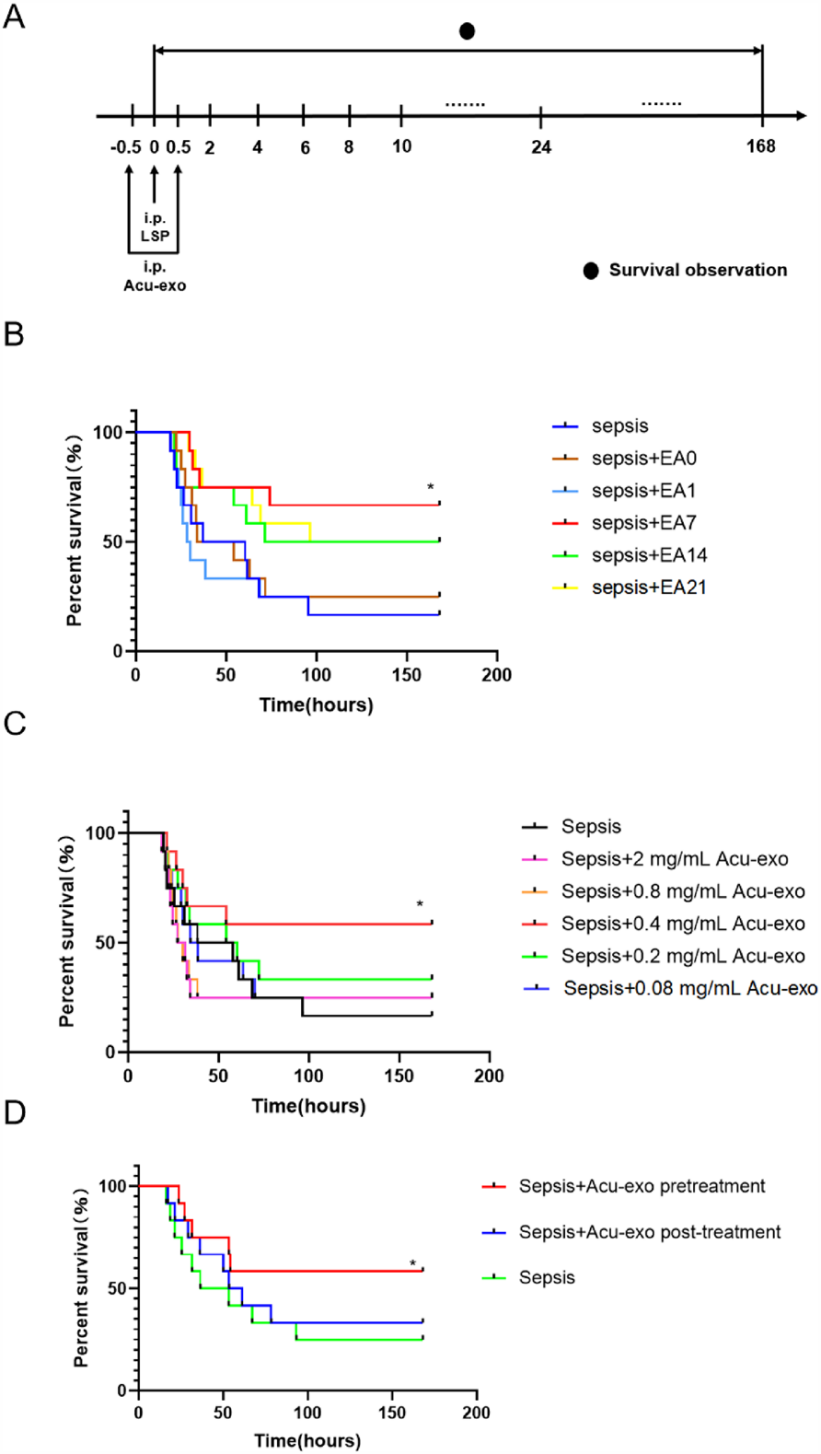
Time-efficacy relationship and concentration-efficacy relationship of Acu-exo. **A** Experimental procedures. **B** Effects of exosomes (0.4 mg/mL) extracted from the circulating serum of normal mice treated with EA0, EA1, EA7, EA14, EA21 on the survival rate of sepsis mice. Acu-exo (0.1 mL/10 g, i.p.) should be administered 0.5 h before the LPS injection. **C** Effects of different concentrations (2 mg/mL, 0.8 mg/mL, 0.4 mg/mL, 0.2 mg/mL, 0.08 mg/mL) of exosomes extracted from the circulating serum of normal mice treated with EA7 on the survival rate of sepsis mice. Acu-exo (0.1 mL/10 g, i.p.) should be administered 0.5 h before the LPS injection. **D** Effect of different injection times of exosomes (EA7, 0.4 mg/mL) on the survival rate of sepsis mice. Acu-exo (0.1 mL/10 g, i.p.) should be administered either 0.5 h before or after the LPS injection. LPS, 12 mg/kg, i.p., n = 12/group. All data represent the mean ± SEM. *P < *0.05*, compared to the sepsis group

Exosomes extracted from the circulating serum of normal mice treated with EA for different days were intraperitoneally injected into sepsis mice. Compared with the sepsis group, the survival rate of the sepsis + EA7 group showed a significant increase (*P* < *0.05*), while there was also an increased survival rate in the other four groups (sepsis + EA0, sepsis + EA1, sepsis + EA14, sepsis + EA21) with no statistical difference (Fig. 3B). Compared with the sepsis group, the survival rate of the sepsis + 0.4 mg/mL Acu-exo group showed a significant increase (*P* < *0.05*), while there was also an increased survival rate in the other four groups (sepsis + 2 mg/mL Acu-exo, sepsis + 0.8 mg/mL Acu-exo, sepsis + 0.2 mg/mL Acu-exo, sepsis + 0.08 mg/mL Acu-exo) with no statistical difference (Fig. 3C). Compared with the sepsis group, the survival rate of the sepsis + Acu-exo pretreatment group showed a significant increase (*P* < *0.05*), while there was also an increased survival rate in the sepsis + Acu-exo post-treatment group with no statistical difference (Fig. 3D). Our results showed that exosomes extracted from the circulating serum of normal mice treated with EA7 (0.4 mg/mL) had the greatest effect on the survival of sepsis mice.

**Fig. 4:**
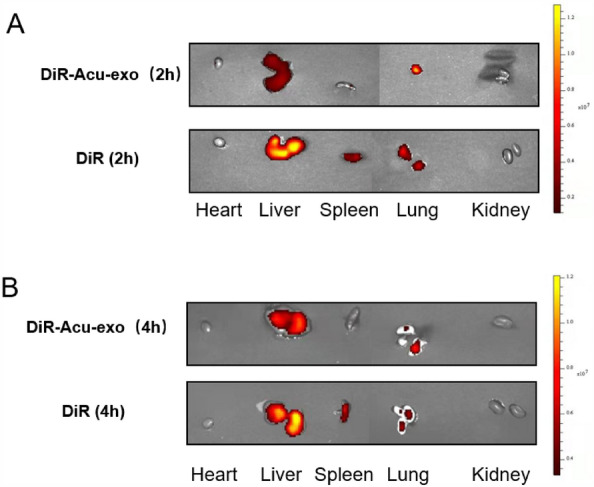
The accumulation in various organs of DiR-Acu-exo. **A** Observe the accumulation of various organs 2 h after DiR-Acu-exo (EA7, 0.4 mg/mL, 60 μL) tail vein injection. **B** Observe the accumulation of various organs 4 h after DiR-Acu-exo (EA7, 0.4 mg/mL, 60 μL) tail vein injection. The injection of DiR or DiR-Acu-exo should be administered 0.5 h after the LPS (12 mg/kg, i.p.) injection

The fluorescent agent DiR or DiR-Acu-exo was injected into the tail vein, and its distribution was observed using in vivo imaging of small animals. Compared with the DiR group, DiR-Acu-exo were more accumulated in the lungs (Fig. 4).

### Comparison the therapeutic effect of EA with Acu-exo in sepsis mice

**Fig. 5:**
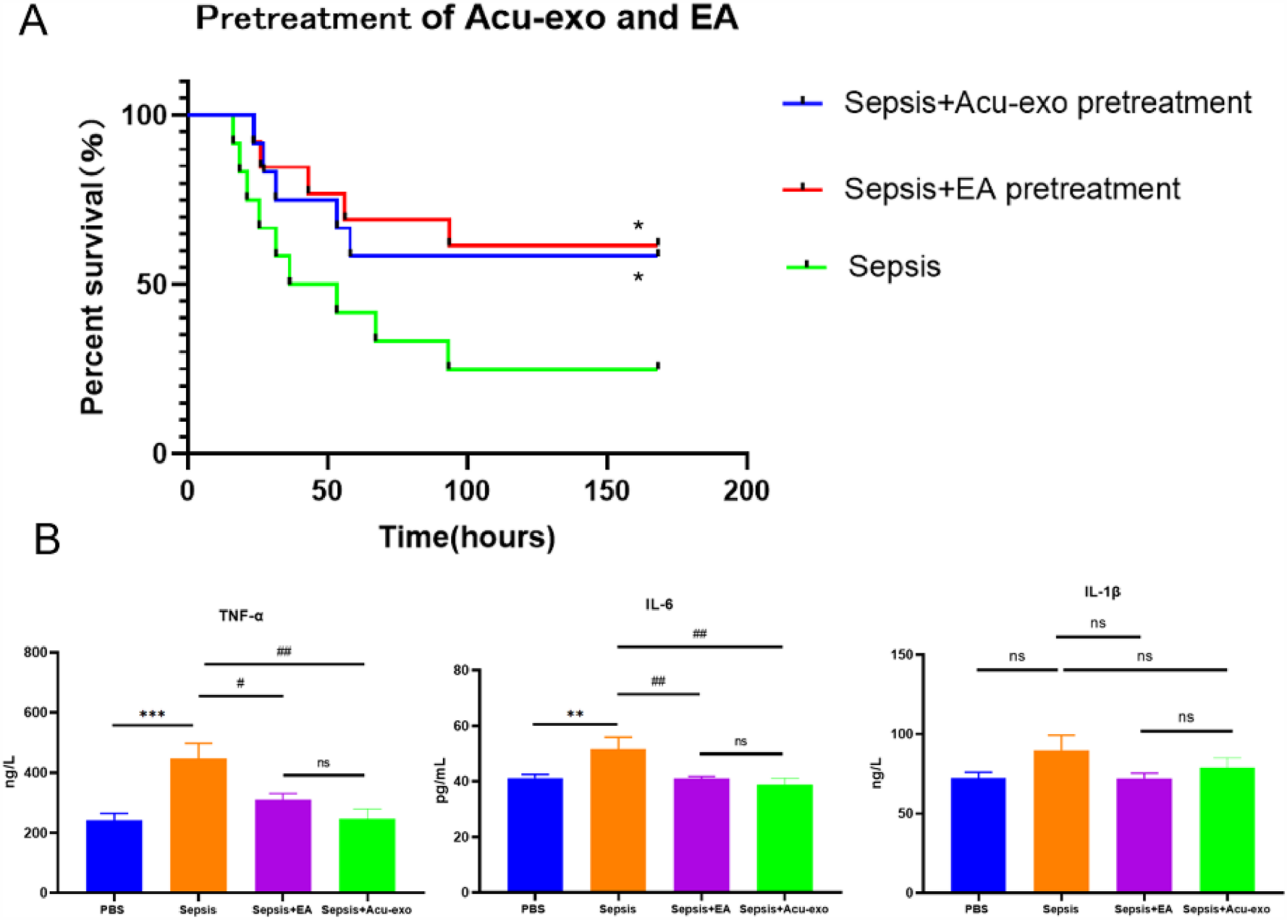
Effects of EA and Acu-exo on the survival rate and serum inflammatory cytokines of sepsis mice. **A** Survival rate within 7 days in LPS-induced sepsis mice (LPS, 12 mg/kg, i.p.) (n = 12/group) (*P<0.05, compared to the sepsis group). **B** Expression levels of serum inflammatory cytokines TNF-α, IL-6 and IL-1β at 2 h after LPS challenge (LPS, 24 mg/kg, i.p.) (n = 5/group) (**P < 0.01, ***P < *0.001*, compared to the PBS group. *#P<0.05*, *## P* < *0.01*, compared to the sepsis group). Acu-exo (EA7, 0.4 mg/mL, 0.1mL/10g, i.p.). All data represent the mean ± SEM

**Fig. 6:**
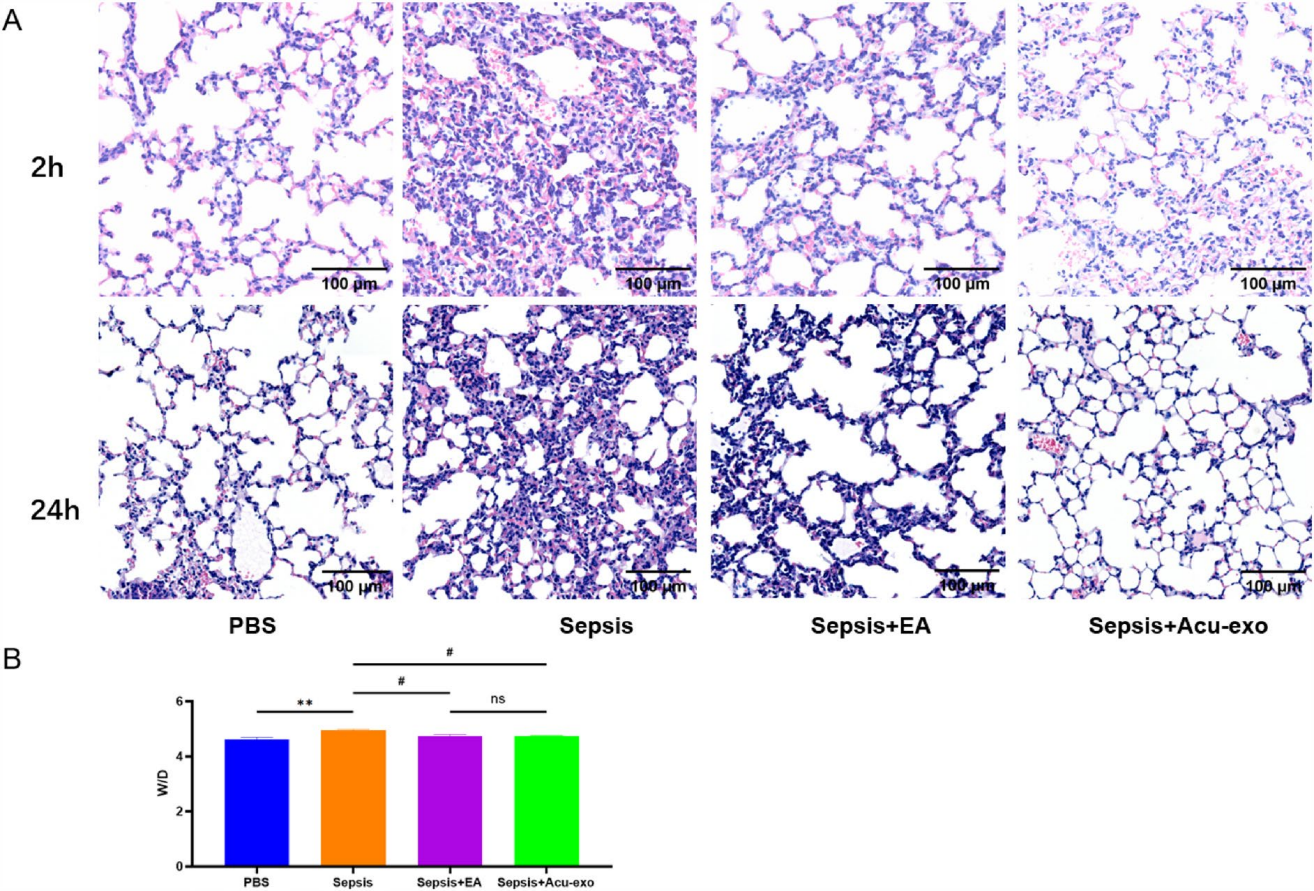
Effects of EA and Acu-exo on lung tissue injury and lung wet/dry weight ration (W/D) in sepsis mice. **A** HE staining of lungs from mice at 2 h and 24 h after LPS challenge. All pictures were taken at 200 × magnification. **B** Lung wet/dry weight ration (W/D) from mice at 2 h after LPS challenge() (n = 5/group). Acu-exo (EA7, 0.4 mg/mL, 0.1mL/10g, i.p.). LPS (24 mg/kg, i.p.). **P < *0.01*, compared to the PBS group. #*P* < *0.05*, compared to the sepsis group. All data represent the mean ± SEM

EA and Acu-exo have similar effects on sepsis mice. Both EA and Acu-exo significantly increased the survival rate of sepsis mice (*P* < *0.05*), with no significant difference between them (Fig. 5A). Both EA and Acu-exo could significantly down-regulated the expression levels of serum inflammatory cytokines TNF-α (*P* < *0.05, P* < *0.01*) and IL-6 (*P* < *0.01*) in sepsis mice, with no significant difference between them (Fig. 5B). Both EA and Acu-exo reduced the infiltration of inflammatory cells and thickening of alveolar wall in the lungs of sepsis mice at 2 h and 24 h after LPS challenge (Fig. 6A). Both EA and Acu-exo significantly reduce W/D of sepsis mice at 2 h after LPS challenge (*P* < *0.05*), with no significant difference between them (Fig. 6B).

### Expression of inflammatory cytokines in LPS-Induced Macrophages RAW264.7

**Fig. 7:**
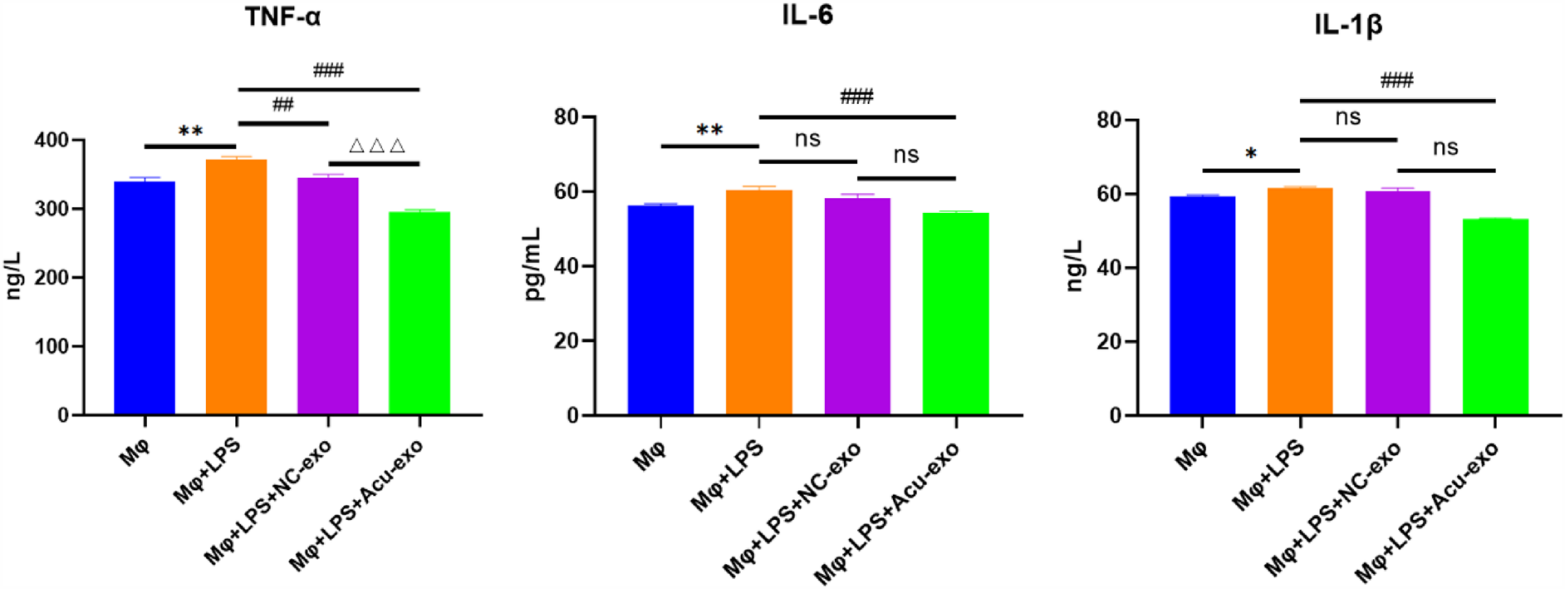
The effect of Acu-exo on the expression of inflammatory cytokines in LPS-induced macrophages RAW264.7. LPS (10 μg/mL), Acu-Exo/NC-Exo (20 μg/mL), n = 3/group. All data represent the mean ± SEM. *P < 0.05, **P < *0.01*, compared to the Mφ group. ##*P* < *0.01*, ###*P* < *0.001*, compared to the Mφ + LPS group. △△△*P* < *0.001*, compared to the Mφ + LPS + NC-exo group

Acu-exo significantly down-regulated the expression of TNF-α, IL-6, and IL-1β (*P* < *0.001*). Although NC-exo also significantly down-regulated the expression of TNF-α (*P* < *0.01*), the effect of Acu-exo was better (Fig. 7).

### MiRNAs carried by Acu-exo may mainly play anti-inflammatory roles in sepsis

**Fig. 8:**
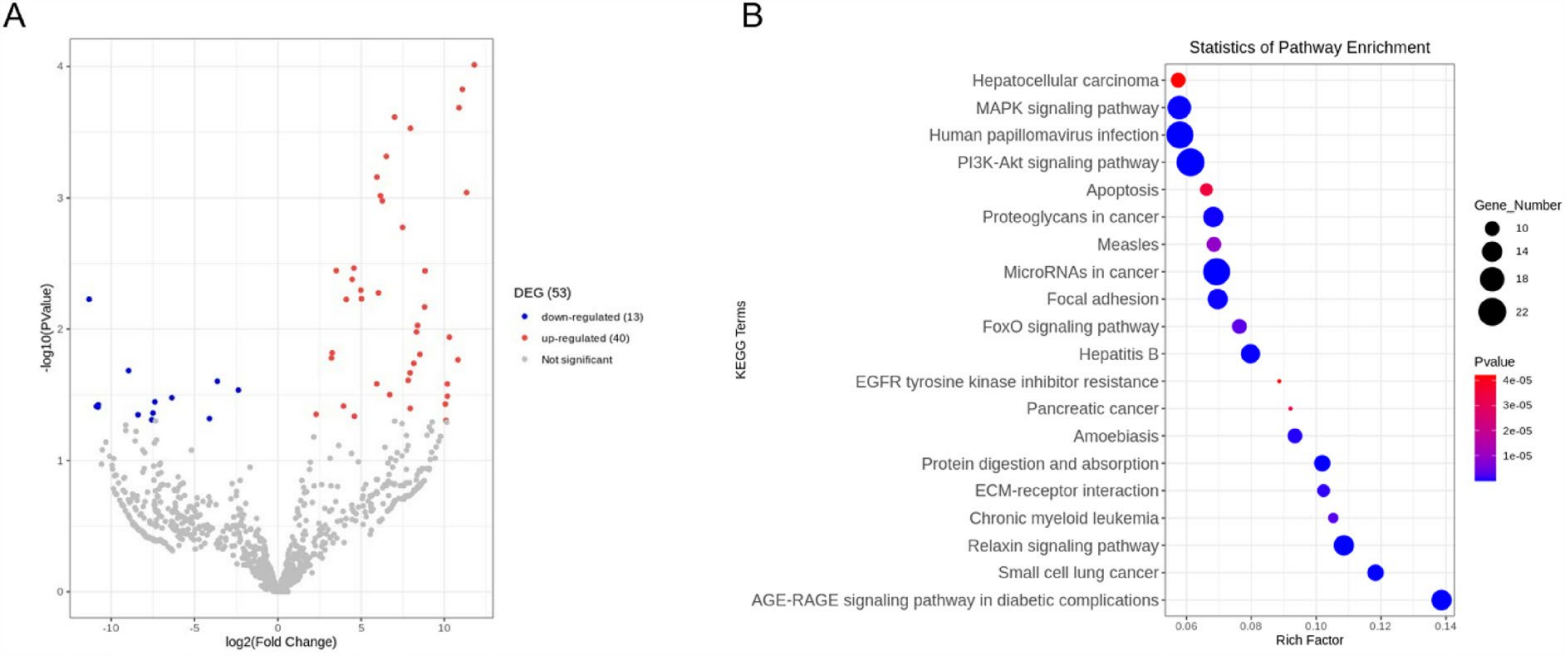
MiRNA sequencing and KEGG pathway analysis. **A** The volcano map showed 53 significantly differentially expressed miRNAs between NC-exo and Acu-exo (n=3/group). **B** KEGG pathway analysis showed the 20 pathway entries with the most significant enrichment. NC-exo group: exosomes extracted from the circulating serum of normal mice. Acu-exo group: exosomes extracted from the circulating serum of normal mice treated with EA7

We used miRNA sequencing to explore the mechanism of Acu-exo in treating sepsis. The results showed that compared with the NC-exo group, Acu-exo group had 53 differential miRNAs, of which 40 miRNAs were up-regulated and 13 were down-regulated (Fig. 8A, Tab.1). KEGG pathway analysis of the differential target genes found that the signaling pathways are mainly enriched in the MAPK signaling pathway, PI3K-Akt signaling pathway, apoptosis signaling pathway, focal adhesion signaling pathway, and FoxO signaling pathway (Fig. 8B). These results suggest that Acu-exo may play an anti-inflammatory role in sepsis by regulating related miRNAs and signaling pathways.

**Table1:** List of differentially expressed miRNAs

Up-regulated miRNAs	Up-regulated miRNAs	Down-regulated miRNAs
mmu-miR-6904-5p	mmu-miR-1964-5p	mmu-miR-1967
mmu-miR-27a-5p	mmu-miR-8091	mmu-miR-1893
mmu-miR-200a-5p	mmu-miR-5129-3p	mmu-miR-129-5p
mmu-miR-101a-3p	mmu-let-7i-5p	mmu-miR-122-5p
mmu-miR-6972-5p	mmu-miR-6993-5p	mmu-miR-8117
mmu-miR-451a	mmu-miR-148a-3p	mmu-miR-6354
mmu-miR-30a-5p	mmu-miR-1933-5p	mmu-miR-130a-3p
mmu-miR-6414	mmu-miR-7027-3p	mmu-miR-9-5p
mmu-miR-215-5p	mmu-miR-6404	mmu-miR-6954-5p
mmu-miR-6941-5p	mmu-miR-3569-5p	mmu-miR-678
mmu-miR-5128	mmu-miR-7010-5p	mmu-miR-7222-5p
mmu-miR-21a-5p	mmu-miR-345-3p	mmu-miR-7082-3p
mmu-miR-206-3p	mmu-miR-1191b-5p	mmu-miR-3091-3p
mmu-miR-1247-3p	mmu-miR-33-3p	
mmu-miR-27b-3p	mmu-miR-362-3p	
mmu-miR-192-5p	mmu-miR-183-5p	
mmu-miR-190b-5p	mmu-miR-29b-3p	
mmu-miR-23a-3p	mmu-miR-143-3p	
mmu-miR-6406	mmu-miR-7008-5p	
mmu-miR-7115-5p	mmu-miR-1927

**Fig. 9:**
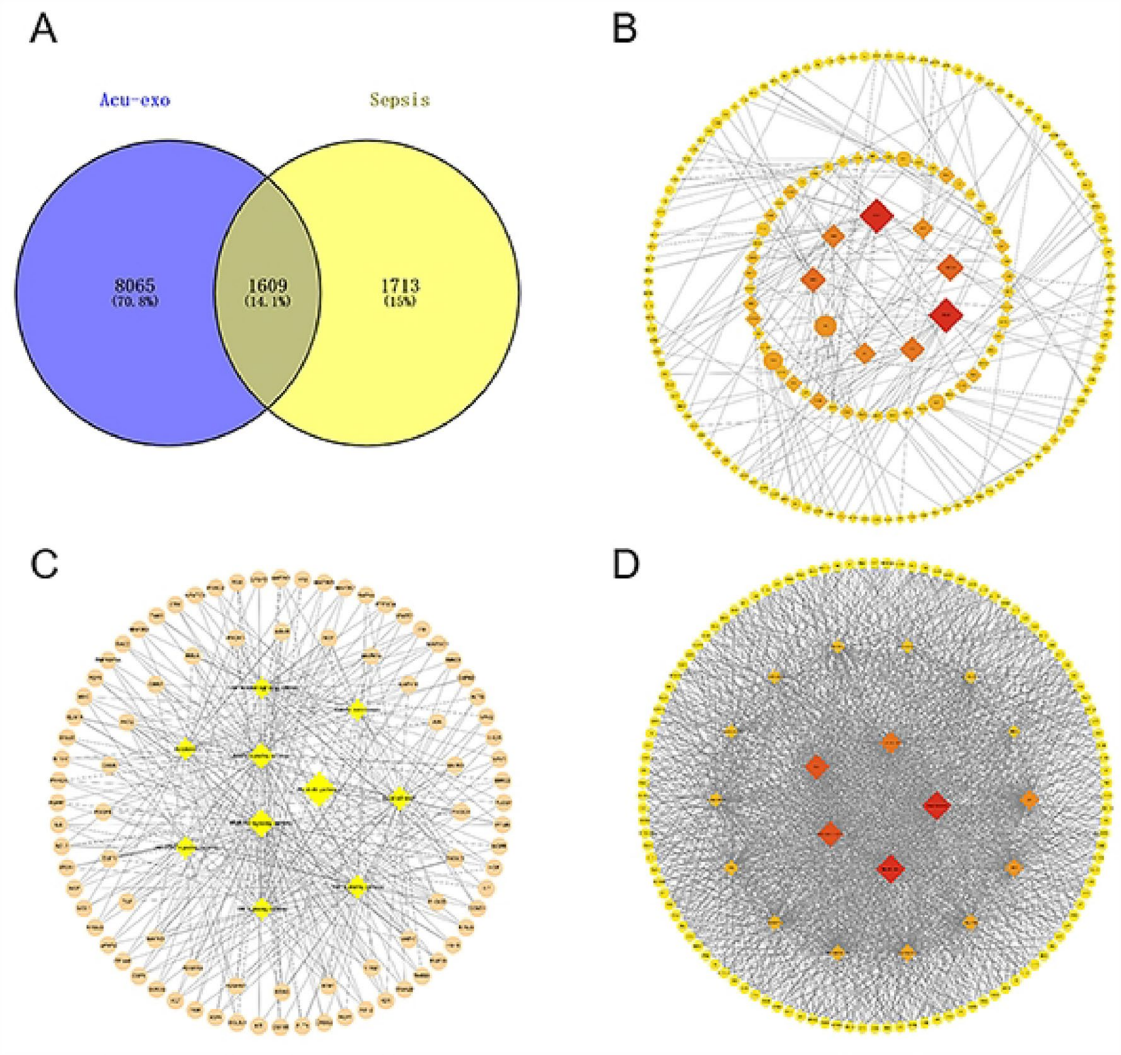
Network biological analysis. **A** Venn diagram of genes co-regulated by Acu-exo and Sepsis (Acu-exo group: mapping differential target genes in miRNA sequencing to human genes, Sepsis group: genes associated with sepsis in the Human Disease Database). **B** Network topology map of interacting genes. **C** Network topology of signaling pathways. **D** Network topology of genes-source. **B-D** Diamonds represent interacting genes, signaling pathways and sources, and circles represent co-regulated genes. The color from yellow to red and sizes from small to large represents the degree value from small to large, a higher Degree indicates a closer connection between them

The Venn diagram showed that there was a total of 1609 human genes with co-regulation of Acu-exo and Sepsis (Fig. 9A). They may be important targets of Acu-exo for the treatment of sepsis.

Among the 20 interacting genes screened, 9 genes were related to inflammation and immunomodulatory, such as heat shock protein 90kDaα (HSP90AA1), Growth factor receptor binding protein 2 (GRB2), and Epidermal growth factor receptor (EGFR). 6 genes are related to tumor immunity, such as histone acetyltransferase P300 (EP300), CREB-binding protein (CREBBP), Catenin 1 (CTNNB1), and the proto-oncogene (JUN). 5 genes were associated with cell motility, proliferation, differentiation, migration, and apoptosis, such as Nonreceptor protein tyrosine kinases (SRCs), Integrin Beta 1 (ITGB1), and Ras homolog family A (RHOA) (Fig. 9B).

Among the 10 signaling pathways screened, Metabolic pathways ranked first, followed closely by signaling pathways related to cell proliferation, growth, senescence and apoptosis such as the PI3K-Akt signaling, Focal adhesions, and the Rap1 signaling pathway. There were also signaling pathways closely related to inflammation and immune regulation, such as MAPK signaling pathway, TNF signaling pathway, JAK-STAT signaling pathway, T cell receptor signaling pathway (Fig. 9C).

Among the 20 sources screened, seven systems and five organs were identified as being involved. Seven systems: Urogenital system, Central nervous system, Respiratory system, Immune system, Cardiovascular system, Skeletal system, and Muscular system. Five organs: Brain, Liver, Gastrointestinal tract, Lung, Kidney. This is in line with the multi-system and multi-organ disease characteristics of sepsis, indicating that genes carried by Acu-exo originates from multi systems and organs (Fig. 9D).

## Discussion

It is well known that the anti-inflammatory effect of EA has been widely studied and recognized. In our study, EA reduced the W/D and lung tissue damage in sepsis mice, down-regulated the expression of serum inflammatory cytokines TNF-α and IL-6, and increased the survival rate of sepsis mice. This is consistent with previous research [8]. Aco-exo was a new intervention method based on serum pharmacology and might be more suitable for studying the mechanism of complex interventions such as acupuncture. Our results showed that Acu-exo (EA7, 0.4 mg/mL, 0.1 mL/10 g) can achieve similar efficacy to EA. Acu-exo has the potential to translate acupuncture into “Acupuncture network durg” [19].

We found that Acu-exo may have a specific targeted effect on diseased organs, as they carried acupuncture information. Lung injury is one of the earliest and most serious complications of sepsis, and it is also an important secondary factor leading to failure of other organs. It accounts for 60% ~ 89.5% of sepsis incidence and has a fatality rate of 30% ~ 40% [22]. Our result found that Acu-exo was accumulated in the lungs, which may be related to integrins on the surface of exosomes. There is an integrin called “zip code” on the surface of exosomes, which enables them to accumulate in specific organs [23]. In addition, some studies have found that acupuncture also have the effect of targeting lesion sites, such as promoting local drug concentration in tumors by regulating tumor microvessels and microenvironment [24]. We believe that Acu-exo may carry acupuncture information and transport it to the site of lesion, so as to play an anti-inflammatory role in sepsis.

Our further cell experiments found that Acu-exo could significantly reduce the expression of LPS-induced macrophage RAW264.7 inflammatory cytokines (TNF-α, IL-6 and IL-1β). Macrophages are divided into M1 and M2 types according to different functions. The M1 is characterized by the release of inflammatory cytokines (TNF-α, IL-6, IL-1β, etc.). Macrophage polarization plays an important role in the development of sepsis. In the early stages of sepsis, macrophages are polarized towards M1 to aggravate inflammatory response and lung injury [25]. Exosomes identified as modulators of the immune system in sepsis. Exosomes derived from adipose stem cells could reduce the expression of IL-1β, TNF-α and IL-6 in macrophages during sepsis while also downregulating M1 markers (iNOS and CD86) expression [26]. Therefore, we believe that Acu-exo may have the potential to regulate macrophage polarization.

MiRNAs are a class of non-coding, single-stranded RNA molecules that are about 22 nucleotides in length and are encoded by endogenous genes. They are involved in a series of important processes in life, including cell proliferation, differentiation, migration, and apoptosis. We believe that the anti-inflammatory effects of Acu-exo are partially mediated by miRNAs within exosomes. MiRNA sequencing results showed that EA altered the expression of multiple miRNAs in circulating serum exosomes of normal mice. Most of the differential genes have the functions of inhibiting inflammation. Among the up-regulated miRNAs, miR-27a-5p reduces the secretion of IL-6, IL-1β and IL-10 in LPS-stimulated macrophages by regulating MCPIP1 overexpression [27]. ADAR1-miR-30a-SOCS3 axis plays a role in reducing inflammation and organ damage in sepsis [28]. LINC00707 attenuates LPS-induced inflammation and apoptosis in PC-12 cells by targeting miR-30a-5p/neurod1 [29]. MiR-215-5p plays a protective role in the inflammatory injury of septic H9c2 by targeting ILF3 and LRRFIP1 [30]. Curcumin protects BV2 cells from LPS-induced damage by regulating the miR-362-3p/TLR4 axis [31]. Among the down-regulated genes, down-regulation of miR-122-5p in Huh7 cells suppressed exosome-induced macrophage activation and macrophage-related inflammation [32]. Inhibiting miR-122-5p can alleviate sepsis-induced myocardial injury by targeting GIT1 to inhibit inflammation, oxidative stress and apoptosis [33]. We further applied network biology analysis to search for important genes that Acu-exo might regulate in sepsis. The genes co-regulated by Acu-exo and sepsis were derived from multiple organs and systems, their interacting genes(HSP90AA1, GRB2, EGFR etc.) and signaling pathways(MAPK, TNF, JAK-STAT etc.) were involved in inflammation, immune regulation, metabolism, and cell proliferation and apoptosis. Therefore, we believe that Acu-exo has great potential in the treatment of sepsis.

There are some limitations in this study. The placebo effect of acupuncture has been a hot topic of discussion, but the experimental design did not include sham electroacupuncture. In addition, we observed the phenomenon of targeted enrichment of exosomes to the lesion site, but the specific mechanism has not been elucidated. Furthermore, miRNAs should be validated in future studies.

## Conclusion

In conclusion, we have discovered that Acu-exo has time-efficacy relationship and concentration-efficacy relationship in treating sepsis. Acu-exo and EA have similar effects on sepsis. The anti-inflammatory mechanism of Acu-exo may be related to the miRNAs carried by Acu-exo and its regulation of macrophage polarization. Endogenous circulating exosomes, driven by EA, may carry multiple targets for sepsis treatment. Translating Acu-exo into “Acupuncture network drug” with broad application prospects can enhance the level of acupuncture application, providing new ideas and approaches for the translational medicine in clinical application of Traditional Chinese Medicine.

## Data Availability

The data used to support the findings of this study are available from the corresponding author upon request.

## Abbreviations

Acu-exo: Acupuncture Exosome
CREBBP: CREB-binding protein
CTNNB1: Catenin 1
EA: Electroacupuncture
EGFR: Epidermal growth factor receptor
ELISA: Enzyme-linked immunosorbent assay
EP300: Histone acetyltransferase P300
GRB2: Growth factor receptor binding protein 2
HSP90AA1: Heat shock protein 90kDaα
ITGB1: Integrin Beta 1
JUN: The proto-oncogene
LPS: Lipopolysaccharide
NTA: Nanoparticle tracking analysis
PBS: Phosphate buffered saline
RHOA: Ras homolog family A
SRCs: Non-receptor protein tyrosine kinases
ST36: Zusanli acupoint
TEM: Transmission Electron Microscopy
